# HIV prevention among transgender women in Latin America: implementation, gaps and challenges

**DOI:** 10.7448/IAS.19.3.20799

**Published:** 2016-07-17

**Authors:** Alfonso Silva-Santisteban, Shirley Eng, Gabriela de la Iglesia, Carlos Falistocco, Rafael Mazin

**Affiliations:** 1 Center for Interdisciplinary Research on Sexuality, AIDS and Society, Cayetano Heredia University, Lima, Peru; 2 Regional Support Team for Latin America, UNAIDS, Panama City, Panama; 3 Horizontal Cooperation Technical Group for Latin America and the Caribbean, Buenos Aires, Argentina; 4 HIV and Sexually Transmitted Infections, Pan American Health Organization, Washington, DC, USA

**Keywords:** transgender women, HIV prevention, prevention, Latin America, PrEP

## Abstract

**Introduction:**

Transgender women are the population most vulnerable to HIV in Latin America, with prevalence between 18 and 38%. Although the region has improved antiretroviral coverage, there is an urgent need to strengthen HIV prevention for key populations to meet regional targets set by governments. We conducted an assessment on the state of HIV prevention among transgender women in Latin America.

**Methods:**

We conducted a desk review of Global AIDS Response Progress Reports, national strategic plans, technical reports and peer-reviewed articles from 17 Latin American countries published through January 2015. The review was preceded by 12 semi-structured interviews with UNAIDS and Pan American Health Organization officers and a discussion group with transgender women regional leaders, to guide the identification of documents. We assessed access to, implementation and coverage of programmes; legal frameworks; community participation; inclusion of new strategies; and alignment with international recommendations.

**Results and discussion:**

Overall, prevention activities in the region focus on condom distribution, diagnosis of sexually transmitted infections and peer education, mostly delivered at health facilities, with limited community involvement. Argentina and Uruguay have implemented structural interventions to address social inclusion. Argentina, Brazil and Mexico have adopted early initiation of antiretroviral therapy and treatment as prevention strategies. The other countries do not have substantial tailored interventions and consider the trans population a sub-population of men who have sex with men in data collection and programme implementation. Limited coverage of services, discrimination and a deep-seated mistrust of the health system among transgender women are the main barriers to accessing HIV prevention services. Promising interventions include health services adapted to transgender women in Mexico; LGBT-friendly clinics in Argentina that incorporate community and health workers in mixed teams; task-shifting to community-based organizations; mobile HIV testing; and gender identity laws.

**Conclusions:**

Transgender women in Latin America continue to have limited access to HIV prevention services, which presents a bottleneck for reaching prevention goals and incorporating new prevention interventions. Prevention programmes should be rights-based; offer tailored, holistic interventions; and involve transgender women in their design and implementation.

## Introduction

Globally, transgender women are one of the populations at highest risk for HIV infection. Recent meta-analyses indicate that transgender women are nearly 50 times more likely to acquire HIV than the general population [1]. Some of the highest HIV prevalence rates occur in Latin America, where transgender women are the population most vulnerable to HIV. Their prevalence rates range from 18 to 38% in countries where they have been measured separately from men who have sex with men (MSM) as an epidemiological category. Additionally, studies carried out using the modes of transmission model (a mathematical model developed by UNAIDS to estimate new HIV cases among different populations in a country) in nine Latin American countries show that new infections among transgender women may account for 1 to 10% of all new infections and that the transmission rate can be higher than that of MSM in some countries [2,3].

Transgender women live in a context of social exclusion and are the population most vulnerable to HIV in Latin America. This vulnerability is a result of the complex interaction of risks at the individual level (e.g. condomless receptive anal sex as a common sexual practice, substance use, high number of partners with sex work as their main economic activity), interpersonal risks (e.g. poor condom negotiation skills with partners and clients, high-risk partner pool) and structural factors (e.g. social exclusion, violence, discrimination, few employment options, lack of legal recognition of gender identity) [4–8]. This complexity demands a multisectoral and multidisciplinary response to the epidemic.

The international response to HIV and AIDS has produced a body of recommendations for HIV programme design and implementation at country level, within an international context of decreasing donor funding to respond to the epidemic. One of these is the Investment Framework for the Global Response to HIV, developed by a working group that included organizations such as UNAIDS, WHO, PEPFAR, the World Bank and the Global Fund, among other academic and policy institutions [9]. It consists of evidence-based interventions for prevention, treatment, care and support for people living with HIV and AIDS. The framework includes the following components: basic programmatic activities (e.g. condom distribution, voluntary counselling and testing, behavioural interventions); social and programme enablers (e.g. laws to protect vulnerable populations, stigma reduction, programme communication, research and innovation); and synergy with development sectors (e.g. for access to education or poverty reduction).

In 2014, the WHO published the *Consolidated Guidelines on HIV Prevention, Diagnosis, Treatment and Care for Key Populations* [10]. This document recommends a combination of promising new strategies for prevention, such as pre-exposure prophylaxis and early antiretroviral treatment following an HIV diagnosis, behavioural interventions, increased access to testing, integration of health services and community participation to strengthen legal enabling environments and address the needs of key populations. Furthermore, the 2015 WHO guidelines recommend that all people living with HIV should be offered antiretroviral therapy regardless of their CD4 count [11]. In recent years, the use of the continuum of care model has allowed countries to track gaps in their national responses [12].

Adopted by Latin American countries, UNAIDS’ Fast Track strategy aims to accelerate action to end AIDS as a public health threat by 2030, by achieving the 90-90-90 targets – where 90% of all people living with HIV know their status; 90% of people diagnosed with HIV are on treatment; and 90% of people on treatment have achieved viral suppression [13]. Countries in the region have also committed to reducing new infections and to eliminating stigma and discrimination [14].

National governments outline their HIV responses in national strategic plans (NSPs) and monitor progress in Global AIDS Response Progress Reporting (GARPR, or “progress reports”) documents submitted to UNAIDS every year [3]. NSPs set a framework for the national response, coordinating stakeholders to assess the epidemiological and social HIV situation, identify the most vulnerable populations, define strategies and interventions and allocate resources for their implementation [15]. GARPRs are official documents produced by countries to monitor progress towards their established targets. Civil society and community organizations carry out various interventions that are integrated into public responses or complement them at the community level.

We aimed to understand how Latin American countries design and implement prevention programmes for transgender women, the current reach and impact of these programmes, the level of community participation, and how these responses align with the international recommendations on HIV prevention among key populations.

## Methods

We conducted a desk review to assess the state of prevention programmes targeting transgender women in 17 countries of Latin America. We focused on the legal framework for the rights of transgender people; the design and implementation of programmes, access to and coverage of prevention services; use of new technologies; alignment of strategies to international recommendations; community participation; and best practices. We assessed national prevention strategies, considering combination prevention as a desired standard. The desk review was preceded by key informant interviews with representatives of UN agencies and transgender women leaders to guide the document search.

### Key informants

We held 12 semi-structured telephone interviews with HIV advisors from UNAIDS and the Pan American Health Organization (PAHO) in the region. Informants were requested to 1) identify the main HIV prevention strategies for transgender women implemented in countries; 2) recommend programmatic documents, evaluations or other working documents that should be included in the review; and 3) identify strategies and interventions that could be considered best practices to improve HIV prevention among transgender women. Interviews were recorded and information analyzed descriptively.

In addition, a focus group discussion was held in Panama in October 2014 with 10 transgender women representatives from the REDLACTRANS, a regional network of national community-based organizations (CBOs) representing transgender women and advocating for their rights. Each country was represented by an elected delegate at REDLACTRANS. The participants – who came from Argentina, Bolivia, Chile, Costa Rica, Ecuador, El Salvador, Honduras, Mexico, Nicaragua, Panama, Paraguay and Uruguay – were asked to identify gaps and barriers in prevention services for transgender women, as well as what they considered effective strategies or best practices implemented in their countries. The discussion session was recorded and the information analyzed descriptively.

### Desk review

We assessed the national responses described in HIV/AIDS NSPs and in the GARPR reports submitted by countries to UNAIDS [3,16]. Additional sources included publicly available reports, health surveys and needs assessments carried out in transgender populations by governmental and non-governmental organizations (NGOs) in the region. We used PubMed and LILACS to identify publications assessing the socio-epidemiological context and HIV prevention interventions for transgender women in Latin America.

We analyzed and critically reviewed documents to 1) identify the epidemiological and social context for transgender women (including the existence of laws or norms, social vulnerability); 2) identify prevention strategies focused on transgender women (as a separate population or, where absent, as part of the MSM population); 3) identify best practices reported by countries in GARPRs and programmatic reports, lessons learned and gaps related to the implementation of prevention strategies for transgender women.

Data collection and analysis were carried out between October 2014 and January 2015. We analyzed the information considering the national level for each response and comparing the situation and strategies implemented in the various countries from a regional perspective. We used key informant interviews to identify documents and strategies referred as best practices. The results shown come from the desk review with the exception of the information on limitations and gaps, which also included the input of key informants from REDLACTRANS.

## Results and discussion

We organized the results to show the main components of national responses considering protective legal frameworks, health sector strategies, access to services and incorporation or new recommendations. In addition, we present the main limitations and gaps in service provision in the region and alternative strategies identified in different countries to overcome existing barriers.

### Protective laws and social environments for transgender women

Table 1 shows the countries in Latin America with laws protecting the civil rights of transgender women. Only Argentina, Mexico City and Uruguay have passed gender identity laws, allowing transgender people to change their name and sex in their identity documents, that is, their legal identity. Colombia and Panama have established judicial procedures allowing the change. All countries in South America with the exception of Peru and Paraguay have laws prohibiting discrimination on the basis of sexual orientation and gender identity. Examining the status of protective laws and social environments described in the progress reports, Central America is the sub-region with the most conservative legal framework for LGBT populations. Some countries, such as Costa Rica and Guatemala, have described in their progress reports how conservative groups represent a key obstacle for HIV prevention either by preventing the design and approval of laws and policies protecting the rights of the LGBT population or by obstructing the implementation of health programmes or sexual education programmes that address sexual diversity. In the border zone between Mexico and the United States, religious groups obstruct condom distribution to key populations and harm reduction programmes for intravenous drug users [17]. In Peru, conservative groups successfully lobbied to remove interventions related to sexual diversity and gender identity from the National Human Rights Plan approved in 2014 [18].

**Table 1:** Legislation for transgender women in Latin America

	Gender identity law	Antidiscrimination law
South America		
Argentina	x	x
Bolivia	–	x
Brazil	–	x
Chile	–	x
Colombia	–^	x
Ecuador	–	x
Paraguay	–	
Peru	–	–
Uruguay	x	x
Venezuela	–	x
Central America		
Costa Rica	–	–
El Salvador	–	–
Guatemala	–	–
Honduras	–	–
Nicaragua	–	–
Panama	–^	–
Mexico	x^a^	x

*Note:* Table 1 shows the laws of Latin American countries protecting the civil rights of transgender women. Gender identity laws are laws directed at transgender persons that allow individuals to change their name and sex of preference in their identity documents. Antidiscrimination laws are laws that prohibit discrimination on the basis of sexual orientation and gender identity

a Mexico City

^ Panama and Colombia allows judicial processes for name and sex change.

### Health sector strategies

Table 2 summarizes the health sector's main HIV prevention strategies for key populations including transgender women, according to NSPs and progress reports. Although every country in the region identifies transgender women as a key population for HIV in their progress reports, only Argentina, Brazil, Mexico and Uruguay have implemented substantially tailored interventions for this population. The rest of the countries in Latin America mainly treat transgender women as a sub-population of MSM in data analysis, programme design and implementation.

**Table 2:** Health sector strategies for HIV prevention for MSM^a^ and transgender women in Latin America, as described in national HIV plans and progress reports

	Prevention strategies for key populations
South America	
Argentina	- Condom promotion and distribution, workshops and campaigns - Educational material to promote access to prevention services - HIV testing enhancement: updating of diagnostic algorithms to include rapid tests, communication campaigns and service provision in Centers of Prevention, Counseling and Testing (CePATS) - Stigma reduction: *Consultorios amigables* (“friendly clinics”), training and sensitization of health personnel, communities and journalists - Collaboration with CBOs to implement activities - Comprehensive and tailored health services for the LGBT population
Bolivia	- Condom promotion and distribution at health facilities - HCT and diagnosis and treatment of STIs at reference and surveillance clinics - Work with peer health promotors
Brazil	- Condom and lubricant promotion and distribution - HIV testing enhancement: updating of diagnostic algorithms to include rapid tests (oral and blood), partnerships with CBOs and NGOs, mobile units - Treatment as prevention, test and offer/universal treatment, comprehensive PEP, demonstration studies on PrEP - Comprehensive and tailored health services for key populations
Chile	- HIV prevention information and materials for MSM and trans women - Condom promotion and distribution at health establishments
Colombia	- HIV prevention information material - Condom promotion and distribution
Ecuador	- Condom promotion and distribution at health facilities - HCT and diagnosis and treatment of STIs at reference and surveillance clinics - Work with peer health promotors
Paraguay	- Condom promotion and distribution at health facilities - Work with peer health promotors
Peru	- Condom promotion and distribution at health facilities - HCT and diagnosis and treatment of STIs at reference and surveillance clinics - Work with peer health promotors at health facilities
Uruguay	- Condom promotion and distribution - Collaboration with CBOs to improve care through health services
Venezuela	No information
Central America	
Costa Rica	- Condom promotion and distribution at health facilities
El Salvador	- Condom promotion and distribution at health facilities - HCT and diagnosis and treatment of STIs at reference centres - Community-based work with key populations to strengthen health services
Guatemala	- Condom promotion and distribution at health facilities - HCT and diagnosis and treatment of STIs at reference centres - HIV prevention information material
Honduras	- Condom promotion and distribution at health facilities - HCT and diagnosis and treatment of STIs at reference centres - Work with peer health promotors at health establishments
Nicaragua	- Communication campaigns on HIV prevention - HCT and diagnosis and treatment of STIs at primary care facilities
Panama	- HIV prevention information and materials - Condom promotion and distribution at health facilities - HCT and diagnosis and treatment of STIs at reference centres
Mexico	- Condom promotion and distribution - HCT and diagnosis and treatment of STIs at reference centres - Work with peer health promotors - Comprehensive and tailored health services for MSM and trans women

*Note:* Table 2 shows the main strategies described by countries in NSPs and progress reports for HIV prevention among MSM and/or transgender women

a MSM are included in this analysis because the majority of countries still treat transgender people as a sub-category of MSM in their data analysis and service provision; CBO, community-based organization; HCT, HIV counselling and testing; MSM, men who have sex with men; NGOs, non-governmental organizations; PEP, post-exposure prophylaxis.

Prevention strategies are similar across countries in the region. In general, they are based on the following core elements: 1) information, education and communication activities; 2) condom promotion and distribution, STI diagnosis and control; 3) voluntary HIV counselling and testing (HCT); and 4) work with peer educators. The majority of prevention services are provided at primary care facilities focused on STI diagnosis and treatment, including HCT. Identified HIV cases are referred to treatment facilities, usually general hospitals. In some countries health personnel carry out outreach activities such as information campaigns or testing at social or sex work venues. In countries like Mexico, the largest clinics provide antiretroviral treatment and offer complementary services such as oral and mental health.

Table 3 shows the countries that offer these primary care services for key populations. Those that do not offer these services at these facilities provide them through the regular public health system. There is a gap in prevention interventions that go beyond condom use and individual-level risk reduction.

**Table 3:** Primary care services for STI diagnosis and control among MSM^a^ and transgender women in Latin America

	STI centres for key populations
South America	
Argentina	Friendly clinics/Centers of Prevention, Counseling and Testing (CePATS)
Bolivia	Centers for Information, Surveillance and Referrals (CDVIR)
Brazil	–
Chile	–
Colombia	–
Ecuador	Comprehensive Centers for Sexual Healthcare (CAISS)
Paraguay	–
Peru	STI Referral Centers (CERITS)
Uruguay	–
Venezuela	No information
Central America	
Costa Rica	–
El Salvador	HIV/STI Surveillance and Control Strategy (VICITS)
Guatemala	HIV/STI Surveillance and Control Strategy (VICITS)
Honduras	HIV/STI Surveillance and Control Strategy (VICITS)
Nicaragua	HIV/STI Surveillance and Control Strategy (VICITS)
Panama	HIV/STI Surveillance and Control Strategy (VICITS)
Mexico	HIV and STI Ambulatory Prevention and Care Clinics (CAPASITS), Clínica Especializada Condesa

*Note:* Table 3 shows the countries that have primary care facilities focused on HCT and STI diagnosis and treatment for MSM and transgender women. Acronyms are shown in Spanish as used in official documents

a MSM are included in this analysis because the majority of countries still treat transgender people as a sub-category of MSM in their data analysis and service provision; HCT, HIV counselling and testing; MSM, men who have sex with men.

### Condom and lubricant distribution

As reported in the GARPRs, countries such as Argentina, Brazil and Mexico carry out mass condom purchases and distribution, making them available to key populations through health services, peer distribution at socialization and sex work venues and through CBOs. Elsewhere in the region, condom distribution for key populations occurs mainly in health centres, which have a more limited scope. The trans women key informants mentioned that the number of condoms distributed to health centres in Bolivia, El Salvador and Panama does not satisfy the demand of the population, especially those engaged in sex work. In a context of poverty and where sex work is one of the few employment alternatives, having to buy condoms is a significant limitation to its usage. Information on lubricant distribution is limited, indicating the need for increased access to and availability of lubricants.

### Access to HIV testing

**Figure 1:**
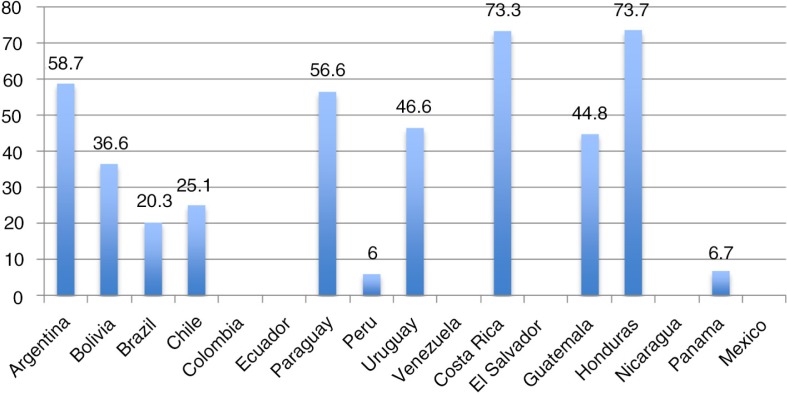
Access to HIV testing among men who have sex with men and transgender women in the past 12 months in Latin America. **Data in the figure are reported by countries in progress reports, as a proxy to measure access to prevention services. Except for Honduras and Panama, countries provided data for men who have sex with men and transgender women as a single population. This classification poses a problem for assessing prevention interventions among different key populations. Countries without data did not report proportions for this indicator in progress reports**.

Figure 1 shows access to HIV testing by MSM in the last 12 months as reported by countries in progress reports. Although it would be more useful to have MSM and transgender women as separate populations, most countries still include data on transgender women within MSM. Nonetheless this indicator is used as a proxy to measure access to prevention services among transgender women. It must be clarified that this indicator is constructed differently across the region, limiting the ability to compare data. Some countries report estimates of programmatic data (users of the service, divided by total estimated programme users), while others estimate the proportion of users divided by the total population. For example, Peru went from using as the denominator the estimated number of prevention programme users to using the estimated total number of MSM/transgender women in the country (i.e. 3% of the total male population between 18 and 45 years). This could explain the coverage rate of 5% shown for this country. In the majority of countries, less than 50% of MSM/transgender women have had an HIV test in the past 12 months. Various epidemiological surveillance studies provide information on transgender women's access to testing and knowledge about HIV status. In a Mexico City study, only 26% of HIV-positive trans women were aware of their status [19], while in Peru this figure was 24% for HIV-positive MSM/trans women [20]. Data across Latin America show that access to testing among transgender women is a major bottleneck for the provision of HIV prevention and care services. It is a critical step to reinforce in the continuum of care.

### Incorporation of new prevention strategies: prevention based on antiretrovirals

The use of antiretrovirals for HIV prevention is still nascent in the region and is currently implemented by three countries. The main strategy consists of HIV testing and the offer to initiate early antiretroviral therapy (test and treat, or test and offer). Brazil began implementing this measure in December 2013 [21] and Argentina and Mexico incorporated it into their treatment guidelines in 2014 [22,23]. Brazil has also established the usage of post-exposure prophylaxis (PEP) for individuals who have engaged in a high risk sexual act (apart from cases of rape) [24]. Although it is available in various settings, no country in the region has incorporated PrEP as a public health strategy for HIV prevention (incorporated as an intervention in national responses). To date, Brazil is implementing two demonstration studies on the acceptability, feasibility and safety of PrEP for key populations [25].

### Health services limitations and gaps

We present the main limitations on the provision of prevention services mentioned in progress reports and identified by transgender women from REDLACTRANS. Several of the limitations mentioned are inherent to the region's public health systems (e.g. accessibility or limited supply). These overlap with issues in the lives of transgender women such as social exclusion and discrimination, which increase their vulnerability and diminish their engagement in prevention services.

#### Geographical accessibility

STI clinics are often limited to one or two per city, reducing their accessibility due to distance and time constraints.

#### Opening hours

In many cases the daytime hours are not suitable for transgender women who engage in sex work or even for those who have formal work during the day. Countries such as Argentina, Honduras and Ecuador identify this limitation in their NSPs and raise the need to tailor schedules to the population's needs.

#### Limitations of the supply chain to ensure availability of drugs and supplies

This issue affects the health system as a whole and is a barrier not only for prevention services (absence or shortage of rapid tests, limited number of condoms for distribution), but also for HIV treatment (documented episodes of ARV stock-outs in several countries in the region).

#### Prevention and care services with no or limited integration

Once a new case of HIV is identified at a primary care facility, the referral to treatment centres (often hospitals) places the onus almost entirely on the client. Information systems are not prepared to track an individual through the entire continuum of care and usually do not address gender identity as a variable to document service provision for a transgender women.

Regarding the quality of services, the following limitations were identified.

#### Lack of protocols on transgender health

The clinics providing HIV services may be the only contact transgender women have with health services, as a population with a high degree of social exclusion. There were few services identified for mental health, survivors of violence and referrals to other public sectors such as justice. Counselling tends to focus mainly on aspects of sexual behaviour. As described below, there are some initiatives seeking to fill this gap. PAHO has developed guidelines for the provision of comprehensive care for transgender persons, including aspects such as gender-affirming processes and hormone therapy [26]. Countries like Argentina have developed comprehensive care guidelines for transgender health [27].

#### Discrimination in health facilities

Discrimination is often caused by the lack of training of health personnel on sexual diversity and transgender health [28]. These situations include non-recognition of gender identity (e.g. use of the male name), stigmatization (e.g. focusing only on HIV or STIs, even if the individual is seeking care for another health problem) and even denial of attention and care [4,5,29,30].

#### Limited community participation and outreach activities

In general, community participation is limited to peer-led health promotion at health facilities targeting key populations, where some promoters are part of the staff. Key informants state that this participation is often insufficient or promoters have not been trained to disseminate prevention messages. In many countries, transgender women have not played a significant role in planning and implementation of interventions.

The social exclusion experienced by transgender women, together with the health system limitations mentioned above, perpetuate a *parallel health culture* centred on gender enhancement and sex work. Transgender women sex workers earn more money if they have a more voluptuous body, which can be achieved by the use of fillers. This culture is based on empirical practices, organized by peer referral and includes hormone self-medication with almost no medical follow-up and the extended use of liquid silicone/fillers [4,5]. It has been described in Argentina, Brazil, Mexico, Peru and Central America [5,19,29–31].

### Alternatives and best practices to improve prevention strategies in the region

We found no experimentally evaluated prevention interventions in the scientific literature specifically related to transgender women in Latin America. Through progress reports, key informants from UN organizations and activists from REDLACTRANS, we identified strategies that have been implemented by governments, civil society organizations and/or CBOs to promote social inclusion, enhance access to services and increase community participation. These strategies are presented, showing specific examples that can be adopted by countries in the region to improve the delivery of HIV prevention services for transgender women.

#### Structural interventions to promote social inclusion

A study conducted by the Association of Transvestites, Transsexuals and Transgender Persons of Argentina and Fundación Huésped documented the positive impact of the gender identity law among trans women [32]. Of the 452 study participants, 57% had changed their gender in their identity document within 18 months of the law's approval. Participants reported a reduction in experiences of stigma and discrimination in the public health system, educational system and with security forces. Changing one's identity was associated with an increase in transgender people returning to the educational system and seeking formal work. Nonetheless, access to the benefits of the law has not been homogenous among transgender women: those who were sex workers and had undergone gender-affirming processes were more likely to have changed their gender identity. Immigrants were less likely to change their identity, highlighting migration as another vulnerability factor faced by transgender women. The data suggest that those who are more empowered were the first to access the benefits of the law [33]. Argentina has also implemented labour inclusion programmes for transgender persons [34].

Uruguay has focused on increasing social inclusion among transgender women. In addition to the gender identity law, the Ministry of Social Development has implemented the following measures since 2012: gender quotas and labour programmes including affirmative action for transgender people, access to food assistance programmes for transgender people living in extreme poverty, raising awareness of public servants on issues of transgender identity, implementation of an observatory to document cases of discrimination and human rights violations, and partnerships with academic institutions to improve the understanding of social vulnerability and design interventions [35].

#### Provision of services that meet the population priorities

Clínica Especializada Condesa in Mexico, one of the largest clinics for key populations in the country, provides tailored services for transgender women to encourage access to HIV services. The intervention includes the provision of free hormone therapy and the training of health personnel to provide transgender health services. Within a year of implementation, the clinic had almost 700 transgender patients, with a higher retention in HIV care than other populations; 90% of those who tested HIV-negative were retested every six months. Some transgender women were also treated for complications due to hormone overdosing and the use of fillers [36].

#### Adaptation of existing services to provide tailored care for key populations

*Consultorios amigables* (“friendly clinics”) in Argentina are key population-friendly health clinics that tailor existing services to LGBT health needs, thus welcoming sexual minorities into the public health system. The clinics require existing services in primary care clinics and hospitals to be reorganized: a dedicated multidisciplinary team (including medical providers, nurses, social workers and LGBT community personnel) is hired and trained; day and night-time hours are offered; primary care services beyond HIV are integrated; and linkages are made with other public institutions (e.g. justice, education, social development) [37].

#### Mobile HIV testing to enhance access

Two projects implementing mobile units that offer rapid HIV testing at social and sex work venues in Lima, Peru, and Esquintla, Nicaragua, compared the population attending mobile units to those accessing testing through health facilities [38,39]. In both cities, those tested by the mobile units had a larger proportion of transgender women and first-time testers. In Lima, mobile units received more people who had engaged in transactional sex and had used drugs or alcohol during the last sexual intercourse. This strategy facilitates reaching a high-risk population that does not necessarily go to health centres and offers alternatives to ministries of health to complement the services offered to key populations at health facilities.

#### Partnering with CBOs to provide services

Since 2014 Brazil has been implementing the project *Viva melhor sabendo* (“Live Better Knowing”) through CBOs, offering oral rapid testing to transgender women and other key populations in communities. HIV testing uptake has increased by reaching out to key populations where they are, as many of them, particularly transgender women, do not attend health facilities. The initiative consists of a grant award mechanism implemented by the MoH, which allocates funding to NGOs and CBOs throughout the country. Oral tests are conducted by trained CBO or NGO staff, who also distribute condoms, lubricants and prevention brochures. People diagnosed with HIV are accompanied to the nearest health facility. As of April 2015, the programme had carried out 28,400 tests, nearly half of which were for first-time testers; 19% of those who tested positive were transgender women [40]. The initiative is a model to increase early case detection and linkage to care.

#### Use of rapid tests for HIV diagnosis

The use of rapid tests can overcome barriers to testing, such as long waiting periods or the need for a second visit to obtain results. It can also facilitate the implementation of mobile strategies for diagnosis [41]. Countries such as Argentina, Brazil and Mexico have adapted their HIV diagnosis guidelines to incorporate the use of rapid tests [42]. Although we did not identify assessments on the impact of this strategy in the region, international literature has shown the effectiveness of rapid tests to increase HIV diagnosis [43].

## Conclusions

### Prevention among transgender women in Latin America: rights-based *versus* disease control

With the exception of Argentina, Brazil, Mexico and Uruguay, the approach to HIV prevention among transgender women is focused on disease control without considering the social determinants of the epidemic. The main strategies implemented by governments consist in condom provision, HIV/STI diagnosis and, in some cases, peer-led health promotion. Community involvement in planning and implementation of interventions is very limited. In several countries of the region, the combination of weak public health systems (with limited human resources and gaps in the integration of prevention and care services) and the high level of social exclusion and mistrust of the health system among transgender women results in insufficient coverage and limited access to prevention services. One of the main gaps identified is transgender women's access to testing, with subsequent effects on the detection of new cases and their link to care and treatment. Furthermore, prevention and care services are not integrated in the majority of countries in the region.

There are also differences between the strategies outlined in planning documents and those that are actually implemented. For example, Ecuador's NSP describes a human rights approach in its HIV response, acknowledging health as a right and recognizing the social vulnerability of key populations, including transgender women. However, prevention efforts are still based on conventional strategies such as condom distribution and HIV testing through health services. In many countries, the NSP is not really a multisectoral plan, but a strategy of the ministry of health's national AIDS programme. Although national programmes coordinate with other public sectors, these linkages are not formalized in official strategies.

Argentina, Brazil, Mexico and Uruguay have shown major progress in achieving an integrated HIV response. The national governments of Argentina and Uruguay have driven policies to guarantee the rights of the LGBT population, addressing health issues (including HIV) through a lens of social inclusion. In both countries there are programmes to promote employment of transgender people and provide assistance to transgender people in poverty. The friendly clinics in Argentina have created welcoming spaces within existing health centres, which increase LGBT people's access. Uruguay has sought to improve service provision for transgender persons starting with the municipal health network. Argentina, Brazil and Mexico have adopted a test and offer strategy, albeit with the significant limitation of treatment services that are provided only at health facilities, to which many trans people have limited access.

Brazil and Mexico have advanced in adapting prevention strategies to international recommendations. Besides offering treatment regardless of CD4 count, they implement harm reduction programmes for drug users following international recommendations. Brazil has incorporated the most comprehensive PEP strategy in the region and is currently assessing the use of PrEP as a public health strategy. Brazil's approach highly prioritizes interventions in key populations that account for the bulk of new infections, including transgender women [21]. It also includes structural interventions such as empowering CBOs to carry out HIV testing. However, in recent years Brazil's HIV response has faced setbacks due to a resurgence of religious and conservative groups that take a particular moral standing on LGBT and HIV issues [44].

## Final remarks

Although all Latin American countries recognize transgender women as a key population for HIV on official documents such as strategic plans and progress reports, very few have tailored programmes and interventions specifically for this population. The majority of countries still implement prevention interventions for MSM and transgender women as a single population. Additionally, prevention continues to be predominantly focused on disease control, aiming to reduce risk at the individual level rather than having a rights-based combination prevention approach.

In most countries the programmatic data collected still includes transgender women as a sub-population within the MSM category, which presents an immense obstacle for the design, implementation, monitoring and evaluation of appropriate interventions for transgender women. This obstacle should be noted as a major constraint in national responses. The paucity of data on transgender women in general is a cause and a consequence of the lack of effective responses to this population. Data on transgender men are nearly non-existent, highlighting a major gap in our knowledge of how HIV affects this sub-group. Additionally there are very few existing indicators to assess the performance of prevention programmes in the region.

Most prevention strategies for key populations are provided at the level of primary care services and focused on STI diagnosis and control. In many cases, these clinics constitute the main, and sole, point of entry to the health system for transgender women. However, there is a very limited offering of transgender-specific health services at these facilities, which, when compounded by discrimination faced in health settings, creates significant barriers to accessing HIV prevention, especially HIV testing. Interventions such as training and raising awareness of health providers and decision-makers are key to improve the delivery of services. At the same time, it is necessary to increase community participation to tailor interventions and implement activities to improve their coverage and effectiveness [45–48].

Access to testing is the main bottleneck in the continuum of care in the region and can be a setback for the implementation of new prevention technologies such as PrEP, which requires that users have continuous access to testing. In the same way, the implementation of test and treat or test and offer requires strengthening of existing public health and community systems with adequate human resources, efficient supply chains, interventions that can identify and reach the populations most at risk with adequate coverage, integration of prevention and treatment services, and an integrated information system across the continuum of care.

To meet the ambitious prevention targets adopted by the region, a multisectoral response is needed, based on human rights and addressing social determinants such as exclusion (including exclusion from health services), stigma and discrimination. Health services must be adapted to the specific needs of the transgender population – and other key populations – and consider their comprehensive health needs, beyond HIV. Healthcare workers need adequate training to be able to address the specific vulnerabilities of transgender women. This assessment has allowed us to identify promising and alternative strategies to overcome these barriers, from the national to the local level and with the active collaboration of the transgender community, which can serve as an example to other countries in the region. Countries must seek to adapt the international recommendations and the best practices described above to their context.
